# Enactment versus Observation: Item-Specific and Relational Processing in Goal-Directed Action Sequences (and Lists of Single Actions)

**DOI:** 10.1371/journal.pone.0099985

**Published:** 2014-06-13

**Authors:** Janette Schult, Rul von Stülpnagel, Melanie C. Steffens

**Affiliations:** 1 Institut für Psychologie, Friedrich-Schiller-Universität Jena, Jena, Germany; 2 Institut für Informatik und Gesellschaft, Albert-Ludwigs-Universität, Freiburg, Germany; 3 Fachbereich Psychologie, Universität Koblenz-Landau, Landau, Germany; University of Milan, Italy

## Abstract

What are the memory-related consequences of learning actions (such as “apply the patch”) by enactment during study, as compared to action observation? Theories converge in postulating that enactment encoding increases item-specific processing, but not the processing of relational information. Typically, in the laboratory enactment encoding is studied for lists of unrelated single actions in which one action execution has no overarching purpose or relation with other actions. In contrast, real-life actions are usually carried out with the intention to achieve such a purpose. When actions are embedded in action sequences, relational information provides efficient retrieval cues. We contrasted memory for single actions with memory for action sequences in three experiments. We found more reliance on relational processing for action-sequences than single actions. To what degree can this relational information be used after enactment versus after the observation of an actor? We found indicators of superior relational processing after observation than enactment in ordered pair recall (Experiment 1A) and in emerging subjective organization of repeated recall protocols (recall runs 2–3, Experiment 2). An indicator of superior item-specific processing after enactment compared to observation was recognition (Experiment 1B, Experiment 2). Similar net recall suggests that observation can be as good a learning strategy as enactment. We discuss possible reasons why these findings only partly converge with previous research and theorizing.

## Introduction

It is widely assumed that lists consisting of action phrases such as “peel a banana” or “clap your hands” are remembered better when these phrases are acted out by the participant during encoding as compared to other encoding tasks (e.g., [1], p. 175). Several mechanisms underlying the so-called *enactment effect* have been discussed (see [2], [3], for reviews). One prominent account postulates that motor activity contributes to superior memory after enactment encoding (e.g., [2]). Other explanations rely on the distinction between item-specific (i.e., features of an individual action) and relational (i.e., conceptual or order relations between actions) processing to account for enactment effects (e.g., [3]–[6]). It has been assumed that enactment draws attention to item-specific information, but does not enhance (and may even distract from) processing relational information. Thus, in goal-directed action sequences, where one action step is embedded into a hierarchy of related action steps, other encoding tasks, such as observation, may lead to similar or even better recall levels than enactment.

The goal of the present research was to study the role of item-specific and relational information in the free recall of goal-directed action sequences. We did so relying on several established procedures: First, we examined the contribution of order-relational information to free-recall performance. Second, we applied a multiple-recall technique that allows computing an index of subjective organization. Third, item-specific information was estimated from recognition performance. As a comparison condition to the goal-directed action sequences, we included a single-action condition where action phrases were not connected by an overarching goal. A good control condition for testing effects of enactment is an observation-encoding task: All relevant aspects of encoding are held constant across conditions, but one person enacts phrases, whereas the other observes. Such a comparison condition was used here.

Memory for actions is typically examined by presenting simple verb-object phrases (e.g., “crack an egg”) for a later memory test. Participants either perform an appropriate movement for each phrase during study or, in an observation condition, they watch someone executing the denoted action. Mostly, memory for actions has been examined under conditions in which the enactment of these actions served no purpose beyond execution during study. In addition, study lists were often constructed with the specific aim that actions should be conceptually unrelated to each other (e.g., “peel a banana”, “knock on the table”, and “comb your hair”) or that actions could be organized according to rather artificial criteria, for example the object-based categories (e.g., “pet a cat” and “feed a horse” as exemplars of the object category animals; e.g., [7]). Thus, actions as investigated in most laboratory experiments have been characterized as being oversimplified (see [8]). It remains to be tested whether findings on the enactment effect for such single actions generalize to activities or action sequences in which goals function to organize the actions (see [9]). Specifically, relational information based on goals or the outcomes of sets of actions can be used to reconstruct which action has to be done when. A good encoding of “in-order-to” and “enable relations” [10] may provide efficient retrieval paths for recalling actions within action sequences.

By performing actions participants are forced to process task-relevant features of verb-object phrases. Thereby, enactment draws attention to item-specific information of a phrase's verb and object as well as the verb-object relation (e.g., [2], ). This is why enactment effects are particularly pronounced in recognition tests (e.g., [7], [17]–[22]). Because of drawing the actor's focus of attention towards item-specific information, as a trade-off, enactment typically does not provoke improved processing of relations among action phrases. For example, enactment encoding and observation led to similar levels of category clustering during recall [7]. However, the organization criterion used in that study was the involved object of the learned verb-object phrases. A more appropriate criterion for the organization of actions could be the actions' goal (e.g., to crack an egg and mix it with flour and milk in order to make a batter for pancakes).

Such a criterion was used in a study by Engelkamp and Zimmer [23] who compared free recall and categorical clustering for lists of action sequences consisting of several verb-object phrases in an enactment and a verbal-learning condition. They argued that relational information about the sequence structure is represented in the conceptual knowledge system and therefore spontaneously activated independent of the type of encoding task. Hence, observed differences in recall levels should occur because of differences in item-specific processing. They computed the frequently used Adjusted Ratio of Clustering (ARC) scores [24] which reflect the degree to which actions belonging to the same category (i.e., action sequence) are recalled together. In line with their reasoning, clustering scores did not differ significantly between enactment and verbal learning. In both encoding tasks, retrieval cues such as the goals of action sequences were used to a similar degree to guide recall. Consequently, the authors attributed the observed free-recall advantage of enactment encoding over verbal learning to superior item-specific encoding in the enactment condition. Unfortunately, no control task focusing on action-related information was included in this study (i.e., observation). Thus, at present, we can conclude that enactment encoding, a task that induces a focus of attention on action-related information, does not enhance clustering along an action-based criterion (i.e., the actions' goal) compared to verbal learning, a task that does not necessarily draw attention to action-related information. However, a comparison of clustering according to action sequences after enactment and observation is missing yet.

Another type of relational information is order information. Observation facilitates the processing of order information more than enactment does [18], [25]. Engelkamp and Dehn [18] hypothesized that observing participants use order information strategically during recall. The efficient use of order-relational information during recall should, in turn, result in similar net recall compared to enactment encoding that uses item-specific information more efficiently. For longer lists of actions, the encoding of order information should become too difficult and consequently free-recall performance should again rather be determined by the degree of item-specific processing. Consistent with this reasoning, those authors found similar recall levels for short lists (8 actions) but an enactment effect for longer lists (24 actions). These findings were restricted to lists of single actions with no obvious conceptual relations among phrases. However, there is also some evidence that order information is, in general, not spontaneously used as retrieval strategy during free recall – even for short lists [7], [25].

Against this backdrop, we argue that goal-directed action sequences provide other forms of order-relational information, such as the hierarchical structure, “in-order-to”, and “enable” relations [10]. Increased processing of item-specific information during enactment encoding should draw away encoding resources necessary to understand such structural relations. During observation more encoding resources should be available to encode these relations. In line with this assumption, Steffens [5] found better organization of free recall protocols along the order presented during the study phase after observation than enactment of action sequences. Recall levels were comparable for both encoding conditions, although the sequence consisted of 68 single actions. It seems that the beneficial effects of good relational encoding during observation and the assumed good item-specific encoding during enactment cancelled each other out, resulting in equal net recall.

The reported studies varied in several aspects from each other, making conclusions difficult. For example, participants carried out the actions with real objects during encoding in Steffens' [5] experiments, whereas participants enacted actions symbolically in Engelkamp and colleagues' experiments [7], [18], [23], [25]. For a list of single actions, the presence of objects during encoding did not affect enactment and observation differentially [26]. However, in action sequences, materials (or objects) are sometimes transformed in such a way that input materials are not discernible and new objects come into being (e.g., different ingredients mixed in a batter for pancakes) [9], [27]. Thus, one cannot conclude that the presence of objects does not affect the pattern of findings for action sequences. Therefore, in the current research, all participants studied the actions without objects in order to be able to directly compare findings to those of Engelkamp and colleagues.

Taken together, theories converge in postulating that enactment facilitates the encoding of item-specific information but does not enhance (and may even disrupt) the encoding of relational information. In contrast, observation enhances the processing of order-related information but not item-specific processing. Consequently, if relational and item-specific information provide equally efficient retrieval cues such as in goal-directed action sequences, observation may lead to comparably good recall as enactment. If, however, relational processing requires the generation of meaningful associations between actions during encoding such as in long lists of single actions, free recall should be better for enactment than observation because free-recall performance should mainly be determined by item-specific information [18]. Whether the actions' overall goals are equally efficient retrieval cues after enactment and observation encoding remains to be tested.

In the current research, we contrasted memory for single actions with memory for action sequences. We tested to what degree the relational information provided in these sequences can be used after enactment and observation. In a first study we investigated order-relational processing by analyzing the percentage of action pairs recalled in the study-phase order in free recall (Experiment 1A). An indicator of item-specific processing was collected by analyzing recognition (Experiment 1B). Supplementing the findings of Experiment 1A, in Experiment 2 we applied a multiple-recall procedure in order to more generally measure subjective organization of recall protocols in each experimental condition.

## Experiments 1A and 1B

In Experiments 1A–B, we compared memory for lists of action sequences and lists of unrelated single actions after enactment encoding and observation. The experiments only differed in the type of memory test: Participants either completed a free-recall test (Experiment 1A) or a recognition test (Experiment 1B).

For free recall of lists of single actions, hypotheses were based on Engelkamp and Dehn's reasoning [18]. We assumed that order information is not spontaneously processed in longer study lists of unrelated actions and net recall should be determined by the degree of item-specific processing. Thus, for lists of single actions, we expected low levels of ordered recall for enactment as well as observation encoding and a net recall advantage of enactment over observation (due to item-specific processing).

For action sequences, order-based information (that is, knowing why and when to do what) can guide retrieval. Engelkamp and Zimmer [23] argued that information about sequence structure is available to a similar degree after enactment encoding and other encoding tasks. According to this reasoning we should observe similar levels of ordered recall and an enactment effect in net recall of action sequences. In contrast, Steffens [5] assumed that information about the sequence structure is more efficiently processed during observation than enactment encoding. Thus, following her reasoning we should observe higher levels of ordered recall for observation than enactment. Further, assuming a trade-off between the encoding of item-specific and relational information, net recall could be more similar for enactment encoding and observation.

Additionally, participants who observed someone executing the denoted actions should exploit relational information more efficiently in recall, whereas the recall of participants who carried out the actions during encoding should be determined by item-specific information. We tested the latter assumption by applying recognition tests in Experiment 1B that are particularly sensitive to item-specific information. Replicating previous findings, we expected an advantage of enactment over observation in recognition independent of the type of list structure, that is, for action sequences and single actions.

For both encoding conditions we expected better recall for lists with a salient organizational structure (i.e. action sequences) than lists of single actions [23], [28].

## Method

### Ethics statement

In Germany not all research is evaluated by an institutional review board. Researchers are generally given responsibility for ethical treatment of research participants if harm to participants can be excluded and if they are neither deceived nor do they reveal personal information. These premises were fulfilled in the research at hand. All participants signed informed consent at the beginning of the experiment. The present research was funded by the German Research Foundation (Deutsche Forschungsgemeinschaft, DFG), and the funding agency corroborated that no approval by an institutional review board is needed.

### Participants

One hundred and forty-two students volunteered to participate in exchange for course credit or a small payment. They were randomly assigned to the conditions of Experiment 1A or Experiment 1B (*M_age_*  =  21.8 years, age range: 18–32 years; 78% women), with the restriction that twice as many participants were assigned to Experiment 1A than 1B because of test power considerations. Fewer participants were assigned to the recognition test because previous studies demonstrated a robust enactment effect compared to observation already with small samples (e.g., [18]).

For free recall, in order to detect a conventional large main effect of encoding condition (*f* = .40, [29]) with Type-I-error  =  Type-II-error = .05, a total sample size of *N* = 84 was needed. All power calculations relied on Faul, Erdfelder, Lang, and Buchner [30]. In Experiment 1A, 98 participants completed free recall tests (n = 26 enacted and n = 26 observed the enactment of action sequences, and n = 24 enacted and n = 22 observed single actions).

In order to replicate the recognition enactment effect of *f* = .65 observed by Golly-Häring and Engelkamp [7], given the same error probabilities, a total sample size of *N* = 33 was needed. In Experiment 1B, 44 participants completed recognition tests (n = 10 enacted and n = 9 observed action sequences; n = 13 enacted and n = 12 observed single actions).

### Materials

Lists were carefully constructed so that the to-be-learned materials were held constant between the single-actions and the action sequences conditions. Constant materials are important because previous research has shown that there are reliable differences in the memorability of different action phrases, with the factors that determine memorability unidentified yet [31]. In a pre-study, we collected a pool of 12 action sequences, each comprising 12 typical actions, resulting in a total of 144 single action phrases. As in previous research on the enactment effect, a verb-object-phrase (e.g., to crack an egg) described each single action. The action sequences were chosen from different contexts: construction (i.e., repairing a flat tire, constructing a birdhouse), food preparation (i.e., barbecuing, making pancakes, preparing a pizza, heaving breakfast), cleaning (i.e., cleaning a window, loading a dishwasher), and several others (i.e., painting a picture, sending a package, pulling out a car, getting ready in the morning; all materials are available from the third author upon request). For each action sequence, the single actions were arranged in a natural order by two independent raters. In addition, we made sure that every action phrase could be enacted symbolically and was unambiguous when presented by itself.

For the action-sequence condition, three material sets were generated from the complete pool of actions. Each material set consisted of eight (out of twelve) different action sequences. The eight action sequences were allocated to four study lists, one list for each study-test trial. Thus, each study list consisted of two ordered action sequences from different contexts (resulting in 24 to-be-studied action phrases per study-test trial). The presentation order of both action sequences within a trial was randomized in every experimental session.

For the single-action condition, allocating one action phrase of every action sequence to a different material list resulted in twelve different material lists. These material lists were treated in an identical manner as the action sequences: Eight (out of twelve) material lists were allocated to four study lists (resulting in 24 unrelated single action phrases per study-test trial). One could argue that actions were not completely unrelated in the single-action condition since two actions of each action sequence were used in every study list. However, these relations were not obvious (e.g., “to crack an egg” and “to turn on the oven” from the sequence “making pancakes”) and there were several actions from other sequences between actions of the same sequence.

The presentation order of actions within a material list was fixed, but the order of the two material lists within a trial was randomized for every experimental session. For all experimental sessions materials were combined in such a manner that no noun or verb was used twice across study lists.

For the recognition test in Experiment 1B, a distractor phrase was constructed from every original action phrase by exchanging the verb with one denoting a different action (e.g., “to froth milk” [die Milch aufschäumen] instead of “to pour milk” [die Milch eingieβen]). None of the distractor verbs was used in any original phrases. For each study list, two versions of the recognition test were constructed. Each test consisted of 12 original phrases and 12 distractor phrases matching the excluded original ones (i.e., the same object, but a different verb). The original and distractor phrases were reversed in a second version of the recognition test. The phrases were presented in a random order.

### Procedure

Participants were informed they were to take part in a memory experiment and their main task was to remember as many action phrases as possible for a subsequent memory test. Up to six students were tested in pairs in one experimental session. If a partner was missing, a confederate completed the pair. The data of the confederates was excluded from analyses. One student of each pair was instructed to enact all action phrases symbolically during the study phase. The other was instructed to observe his or her vis-à-vis carefully who would enact each action. The respective encoding task within a pair was assigned randomly and task comprehension was tested in a practice trial.

The procedure for all study-test trials was identical. Using PsyScope software [32], the previously recorded action phrases were presented auditorily at a rate of six seconds per item via a loudspeaker. The experimenter checked whether participants enacted all actions and observed their partner, respectively. After a two-minute nonverbal distractor task participants completed either a free-recall test (Experiment 1A) or an old/new-recognition test (Experiment 1B). Test time for both types of memory test was restricted to two minutes. Then, the next study list was presented. The experiment lasted about 20 minutes.

### Design

Both experiments used a 2 (encoding task: enactment vs. observation) × 2 (list structure: single actions vs. action sequences) design in which both factors were manipulated between participants. Dependent variables in Experiment 1A were free-recall performance and the percentage of pairs recalled in the same order as presented during study (as a measure of order-relational processing). In Experiment 1B, the dependent variable was recognition performance (measuring item-specific encoding).

## Results

For all statistical analyses, the Type-I-error was set at ≤ .05. As an indicator of the effect size, partial R^2^ (R^2^_p_) is reported for statistically significant effects [29]. All data analyses were carried out separately for each memory test.

### Free recall

**Figure 1:**
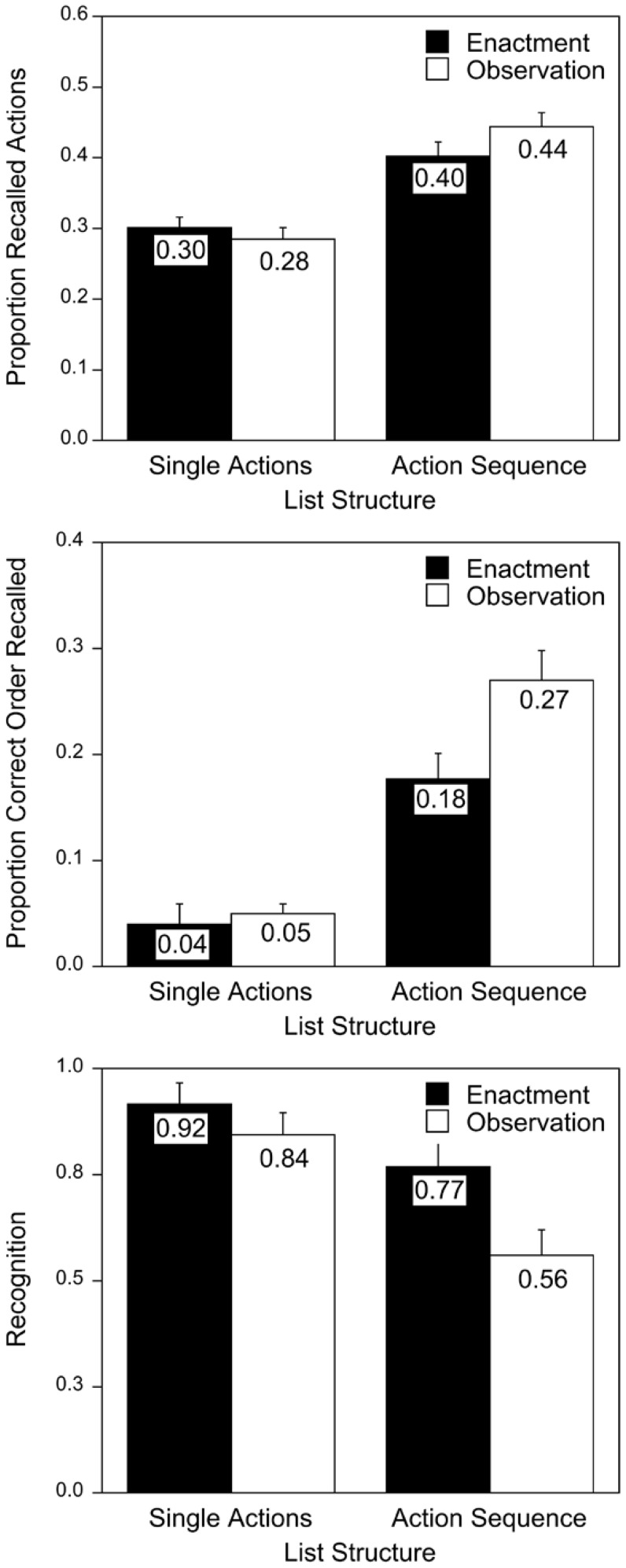
Mean free recall performance (upper) and proportion of ordered recall (middle) in Experiment 1A and recognition performance (bottom) in Experiment 1B, separately for encoding task and list structure. Error bars represent standard errors of mean.

Phrases were classified as correct if original or synonymous nouns and verbs were written down. Use of synonymous nouns mainly occurred because of regional difference in German word use (e.g. “Eierkuchen” and “Pfannkuchen” for pancakes). All analyses were carried out by a computer program, counting once accepted words as correct for all other participants. The pattern of findings is identical if synonyms are excluded. The mean proportions of actions recalled in Experiment 1A are summarized in Figure 1 (upper panel). A 2 (encoding task) × 2 (list structure) ANOVA revealed a main effect of list structure, F(1,94) = 49.84, p<.001, R^2^_p_ = .38. Participants recalled more actions when listening to action sequences (M = .47) than single actions (M = .33). There was no main effect of encoding task, F<1, and no significant encoding task × list structure interaction, F(1,94) = 2.10, p = .15, R^2^_p_ = .02. Whereas visual inspection of Figure 1 suggests better recall of action sequences after observation than enactment, this difference was not statistically significant [simple main effect: F(1,94) = 2.81, p<.10, R^2^_p_ = .03].

### Input-output correspondence

Participants studied only two sequences per trial. Therefore, analyzing input-output correspondence seemed a more sensitive measure of relational processing than a measure of categorical clustering. In addition, for the single-action condition clustering would not be informative since only two random actions of each action sequence were chosen per study list. In Experiment 2 we report different organization scores.

As an index of order-relational processing we computed the percentage of pairs of actions recalled in the same order as presented during study (see [33]) (see middle panel of Figure 1). Although ordered recall was poor in the single-action condition, the score differed from zero (ts>3.90, ps≤.001, for all four conditions). A 2 (encoding task) × 2 (list structure) ANOVA yielded a main effect of list structure, F(1,94) = 77.23, p<.001, R^2^_p_ = .45, a main effect of encoding task, F(1,94) = 5.81, p = .018, R^2^_p_ = .06, and an interaction of both factors, F(1,94) = 4.48, p = .037, R^2^_p_ = .05. Simple main effect analyses showed that participants who observed their vis-à-vis enacting action sequences recalled more actions in the presented order than participants who enacted these sequences themselves, F(1,94) = 10.93, p = .001, R^2^_p_ = .10. In the single-action condition, few actions were recalled in the presented order and the scores did not differ between both encoding tasks, F<1.

### Recognition

Snodgrass and Corwin [34] recommended reporting corrected recognition scores (measured in terms of hits minus false alarms; findings are comparable in both experiments if d' and C are used). These were analyzed in a 2 (encoding task) × 2 (list structure) ANOVA (see lower panel of Figure 1). There was a main effect of encoding task, F(1,40) = 6.49, p = .015, R^2^_p_ = .14, as well as a main effect of list structure, F(1,40) = 15.26, p<.001, R^2^_p_ = .28. As expected, participants in the enactment condition (M = .84) recognized more actions correctly than participants in the observation condition (M = .70). Participants also recognized more actions correctly when studying single actions (M = .88) than action sequences (M = .66). The interaction of both factors was not significant, F<1.6.

For the sake of completeness, we also report results for hits and false alarm rates. Hit rates were higher after enactment than after observation, F(1,40) = 4.25, p = .046, R^2^_p_ = .10, and hit rates were higher for single actions than action sequences, F(1,40) = 13.02, p = .001, R^2^_p_ = .25, The interaction was not significant, F<1 (single-action condition: M = .97, SD = .05, for enactment and M = .91, SD = .05, for observation; action-sequence condition: M = .84, SD = .24, for enactment and M = .69, SD = .23, for observation). False alarm rates did not differ between conditions, all Fs<2.65, ps>.11 (single-action condition: M = .05, SD = .04, for enactment and M = .07, SD = .06, for observation; action-sequence condition: M = .06, SD = .09, for enactment and M = .13, SD = .13, for observation).

## Discussion

For action sequences, the results of recognition (indexing item-specific processing) and input-output correspondence (indexing order-relational processing) support the notion of a differential contribution of item-specific and relational information to net recall after enactment encoding and observation. Replicating Steffens [5], participants who observed the pantomimic enactment of action sequences during encoding exploited the sequence structure more efficiently during recall than participants who enacted the actions. Considering the clear enactment effect in recognition, it is plausible to assume that the comparable net recall for action sequences is the result of increased item-specific processing at the expense of relational processing in the enactment condition and increased relational processing at the expense of item-specific processing in the observation condition.

For the single-action condition, findings are less clear. Following Engelkamp and Dehn's [18] rationale, we had assumed that free-recall performance is mainly driven by item-specific information. Enactment of single actions should enhance the encoding of item-specific information more than observation. The combination of low order-relational processing in both encoding tasks and superior item-specific processing after enactment encoding should result in an enactment effect in free recall (for long lists of actions as were used in this experiment). Indeed, equally poor ordered-recall scores in both study conditions indicate that the presentation order of the study phase was not strategically used during free recall. However, an inspection of the recognition test in Figure 1 suggests that participants in the observation condition processed item-specific information of single actions not much worse than participants in the enactment encoding condition. Thus, comparable net recall for single actions may be the result of similarly good item-specific processing after enactment and observation. Yet, these tentative conclusions about the role of item-specific information in net recall are based on the visual inspection of old-new discrimination in a recognition test with other participants. To gain more evidence about the use of item-specific information in free recall after enactment encoding and observation, in Experiment 2 we used even longer study lists and collected recognition data from the same participants.

A final finding from Experiment 1 was that in free recall, the action-sequence condition with a salient list structure led to better net free recall than the single-actions condition (for similar findings see [23],[28]). In recognition, however, a salient list structure was counterproductive for performance. We address this finding in the General Discussion.

## Experiment 2

The aim of Experiment 2 was to replicate and extend the findings of Experiment 1. Again, we compared memory for lists of blocked action sequences and lists of unrelated single actions after enactment encoding and observation. In addition to free recall and recognition, we tested again whether relational information is used more efficiently after observation than enactment. In order to capture more subtle relations between actions that may not be revealed by sequence clustering, repeated recall tests were used so that subjective organization could be computed: the stability of the recall order over consecutive recalls [35]. It reflects the individually determined sequential order of repeated recalls without prior specification of the source of organization. Participants studied one list of actions followed by three free recall tests.

Another indication of improved organization is an increased net recall over repeated recall tests (see [39], for a review). To the degree that our participants became more and more aware of the sequence structure, increasing net recall should be observed.

For long lists of unrelated actions as used here, if free recall in the observation condition is mainly based on subjective organization between these actions (providing less effective retrieval cues than recall mainly based on item-specific information as in the enactment condition), this should result in a net recall advantage for enactment encoding. Experiment 1A suggests that relations within sequences are used less efficiently after enactment encoding than after observation. We therefore expected more stable recall orders after observation relative to enactment. Again, better recognition performance after enactment than observation was expected independent of list structure.

## Method

### Participants

Similar to Experiment 1A, we aimed at detecting a conventional large main effect of encoding condition in free recall. Given 76 participants, the statistical power to detect an effect of *f* = .40 with a Type-I-error probability of .05 was (1 – Type-II-error  = ) .93. Students volunteered to participate in exchange for candy (M_age_  =  21.6 years, age range: 18–46 years; 77% women). Thirty-six participants were randomly assigned to the single-actions condition (n = 18 for enactment and observation, respectively); 40 participants to the action-sequences condition (n = 20 for enactment and observation, respectively).

### Materials

Six action sequences were chosen from Experiment 1 (repairing a flat tire, barbecuing, making pancakes, loading a dishwasher, painting a picture, sending a package). Sequences were shortened to ten action phrases per sequence. In the action-sequence condition the actions were presented as blocked action sequences but the order of the sequences was randomized in every experimental session. In the single-action condition the same 60 actions were presented in another random order in each experimental session. The recognition test was constructed analogously to Experiment 1.

### Procedure

The general procedure was similar to the previous experiment. Participants studied a list consisting of simple action phrases that were presented auditorily either as ordered action sequences or as single actions in a random order. One participant of each pair enacted these actions symbolically, whereas the other observed their partner. Instead of several short lists, all phrases were presented as one long list. After the study phase participants worked on a short nonverbal filler task. Then, memory was assessed in three consecutive written free recall tests. Recall tests were repeated without an intervening restudy phase. Participants were instructed to write down, in any order, all actions they recalled. After three minutes they were asked to turn the page and immediately do the next free recall. They were encouraged to write down all phrases they recalled, including those remembered during the previous test. Finally, a written recognition test was administered without time limits. The experiment lasted about 30 minutes.

### Design

Again, the factors encoding task (enactment versus observation) and list structure (single actions versus action sequences) were manipulated between subjects. For the analyses of net recall and organization, recall test was treated as a repeated-measures factor.

## Results

### Free recall

**Figure 2:**
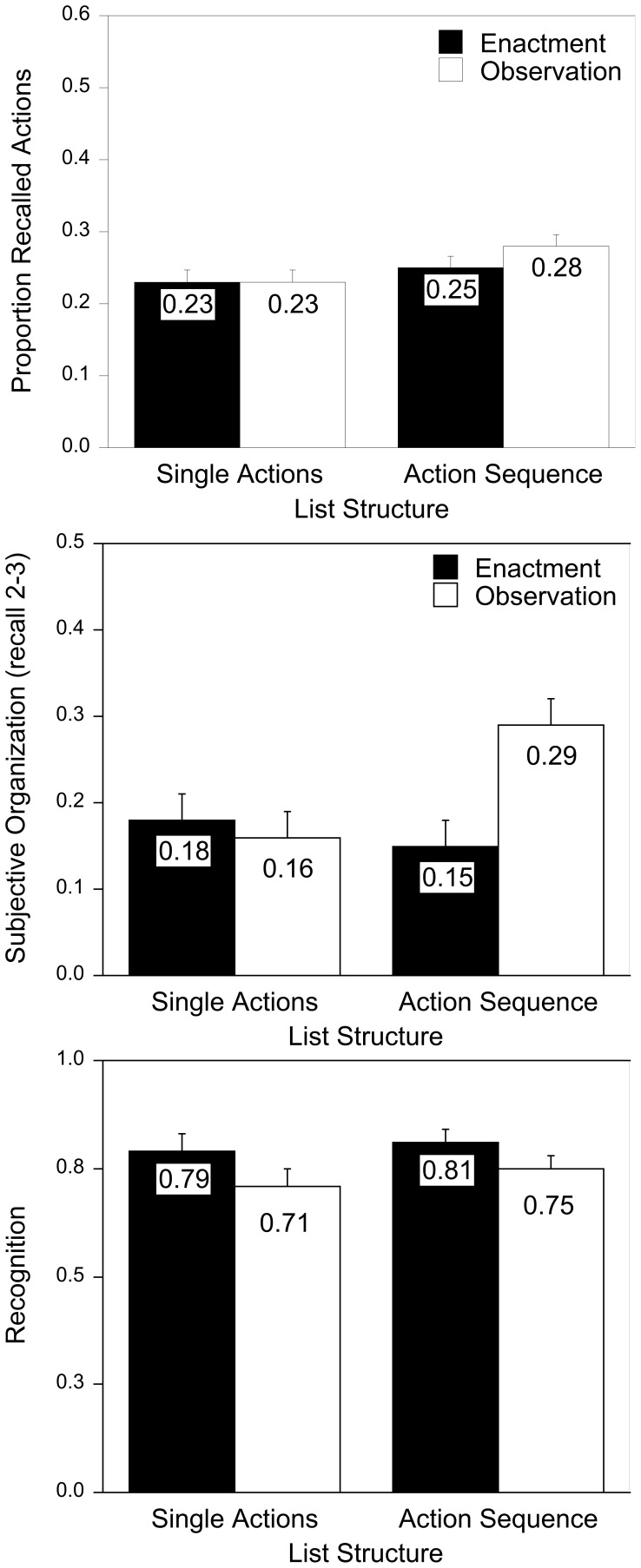
Mean free recall performance (upper), subjective organization scores (middle), and recognition performance (bottom) in Experiment 2, separately for encoding task and list structure. Error bars represent standard errors of mean.

Free recall was analyzed in a 2 (encoding task) × 2 (list structure) × 3 (recall test) mixed ANOVA. The averaged recall probabilities for both encoding tasks and types of list structure are presented in the upper panel of Figure 2. Replicating Experiment 1A, participants recalled more actions presented as action sequences (M = .27) than in a random order (M = .23), F(1,72) = 3.74, p = .028, R^2^_p_ = .05 (one-tailed). Again, neither the main effect of encoding task nor any interactions involving this factor were significant (Fs<1.39, ps>.25). There was also a main effect of recall test (M = .24 for the first, M = .24 for the second, and M = .27 for the third recall test), F(2,144) = 22.06, p<.001, R^2^_p_ = .23. Repeated within-subject contrasts showed that recall levels did not differ significantly between the first and the second test, F<2.5, but participants' recall improved from the second to the third test, F(1,72) = 27.93, p<.001, R^2^_p_ = .28. Recall improvement was not moderated by list structure, F<2.06, p>.13.

### Subjective organization

As a measure of stability of recall order, we calculated subjective-organization (SO) scores as proposed by Tulving [35], reflecting the frequency of sequential occurrence of the same two actions in successive recall runs, irrespective of the order within a pair of actions, relative to maximum possible organization. SO scores were computed for free-recall protocols from recall run 1 to 2, and from recall run 2 to 3. SO scores can range from 0 (no organization) to 1 (perfect organization). A 2 (encoding task) × 2 (list structure) × 2 (consecutive recalls) ANOVA was conducted. Unsurprisingly, subjective organization was higher if action sequences were presented (*M* = .23) compared to single actions (*M* = .16), *F*(1,72) = 6.77, *p* = .01, *R^2^_p_* = .09. Also, the three-way interaction was significant, *F*(1, 72) = 8.81, *p* = .004, *R^2^_p_* = .11. To explore this interaction, separate 2 (encoding task) × 2 (list structure) ANOVAs were computed for each recall run. For recalls 1–2, there were no effects (both *F*s<1) except, again, better organization of sequences than single actions, *F*(1, 72) = 8.74, *p* = .004, *R^2^_p_* = .11 (single actions: *M* = .14 (enactment) versus *M* = .16 (observation), action sequences: both *M*s = .24). For recalls 2–3, there was a significant interaction of encoding task and list structure, *F*(1,72) = 5.69, *p* = .02, *R^2^_p_* = .07 (see Figure 2). Simple main effect tests revealed that SO scores did not differ between enactment and observation of single actions, *F*<1, but the stability of protocols was higher after observation than after enactment of action sequences, *F*(1,72) = 9.27, *p* = .003, *R^2^_p_* = .14.

Originally, we also planned to analyze item gains (actions recalled on later tests but not on earlier ones) and item losses (actions recalled on earlier tests but not on later ones) as additional indicators of organization. There is consensus that good item-specific encoding increases the number of item gains across tests, whereas good relational encoding reduces the number of item losses [36]–[38]. However, our findings indicated that these measures cannot be interpreted when a study list consists of sequences of items. For example, against all expectations, participants who studied action sequences (*M* = .31) lost more items from recall to recall (indicating worse relational processing) than participants who studied single actions (*M* = .23). Inspections of recall protocols showed that this was due to forgetting of whole sequences in the former condition. It thus seems that losses cannot be interpreted as a lack of relational encoding. Because of these anomalies, findings are not reported. We conclude that gains and losses should be interpreted with caution when study lists are structured.

### Recognition

PR scores are presented in Figure 2. Replicating Experiment 1B, participants who enacted actions (*M* = .80) recognized more phrases correctly than participants who observed others (*M* = .73). A 2 (encoding task) × 2 (list structure) ANOVA confirmed this enactment effect, *F* (1,72) = 4.05, *p* = .048, *R^2^_p_* = .05. No other effects were significant, *F*s<1.

Hit rates were higher after enactment than after observation, F(1,72) = 15.21, p<.001, R^2^_p_ = .17 (both other Fs<1.78; single-action condition: M = .93, SD = .07, for enactment and M = .83, SD = .09, for observation; action-sequence condition: M = .90, SD = .10, for enactment and M = .85, SD = .08, for observation). False alarm rates did not differ between conditions, all Fs<1.70 (single-action condition: M = .14, SD = .09, for enactment and M = .12, SD = .13, for observation; action-sequence condition: M = .09, SD = .11, for enactment and M = .10, SD = .08, for observation).

## Discussion

As in Experiment 1A, net recall was comparable for enactment encoding and observation for action sequences as well as single actions. Again, there were hints that observation encourages the processing of relational information as compared to enactment encoding: Better organization in later recalls indicates that structural information was eventually used more efficiently to guide retrieval after observation compared to enactment. It appears that participants needed time or practice to develop retrieval strategies that improve recall performance. Thus, net recall improved from Test 2 to Test 3, and this increase in net recall was accompanied with better organization of the later recall test, but only in the observation, action sequences condition.

Recall performance depended on list structure. Replicating Experiment 1, more actions were recalled when ordered as goal-directed action sequence than when presented in a random order, and recall was organized along the provided list structure of action sequences. In recognition, we found only an enactment effect. In contrast to Experiment 1B, recognition was not better in the single-actions than action-sequences condition.

## General Discussion

The aim of the present research was comparing memory for single actions with memory for action sequences learned by enactment versus observation. When actions are embedded in action sequences, relational information provides efficient retrieval cues; we tested to what degree this relational information is used during recall in both encoding conditions. In contrast, recall of single actions should, in long lists of actions as used here, rather be guided by item-specific processing that has been hypothesized to profit from enactment.

Our findings can be summarized as follows. Comparing enactment and observation yielded several hints for better relational processing of action sequences in the observation condition. First, observation of action sequences yielded better order recall than enactment (Experiment 1A). Second, we found better subjective organization of action sequences in the observation than enactment condition, but only starting from the second recall test. Additionally, relational information was much better exploited in the action-sequences than single-actions conditions. Along with order recall, free recall was increased in the action-sequences compared to the single-actions conditions (both in Experiments 1A and 2), suggesting that free recall was based on relational processing.

Converging evidence of better item-specific processing in the enactment than observation condition was the recognition advantage observed (Experiments 1B and 2). But, in contrast to expectations, free recall of single actions apparently did not profit from this item-specific processing: it was not superior after enactment than observation, neither with relatively long lists (Experiment 1A) nor with very long lists (Experiment 2).

## Implications of the present findings

We had started this research with the assumption that free-recall performance mainly profits from item-specific information after enactment encoding and mainly from relational information after observation. Previous studies often tested the availability of order information after enactment encoding and observation in separate order-reconstruction tests [7], [18], [25] and inferred from those findings which retrieval processes took place during recall. Our experiments provided more direct evidence on the role of relational information during free recall because measures of relational information were computed from the respective free-recall data. Taken together, the present findings support the notion of (somewhat) better relational processing in the observation compared to the enactment condition.

Experiment 2 is the first using multiple recall tests comparing enactment and observation, two tasks that draw attention toward action-related information during encoding. Administering the recall test repeatedly without intervening study trials is ideal for measuring relational processing because scores depend on any type of relational processing, and no assumptions are necessary which type of relational processing participants use (e.g., category clustering, organization by aims, or by input order). Using these measures of subjective organization, we found limited evidence for better relational processing in the observation than enactment condition; had we not included three recall trials, we would have missed this evidence. By implication, memory differences after enactment and observation may unfold only over time, and future research should take this into account when designing respective experiments.

One could speculate whether participants detected the action-sequence structure during the study phase or only later during recall. Actions were presented one after the other without cues indicating the end of one sequence and the beginning of the next one. A repeated recall may have helped participants to detect the possible organization according to action sequences. In a previous study, for enactment encoding knowledge about list organization (before the study phase) did not affect clustering in recall; however, such pre-information was used in a verbal-learning condition, resulting in better clustering and higher net recall [40]. It could be that observation would benefit in a similar way from explicit information about list structure. However, we do not know whether this is the case because in our experiment participants had to find out this information themselves.

For lists of single actions, we had expected an enactment effect in free recall based on superior item-specific processing. Recognition was indeed better after enactment than observation. However, the corresponding enactment effect that we expected in free recall could not be observed. Why could this be?

It should be noted that the majority of studies investigating the enactment effect used a verbal-learning control condition. Most of those studies found significant enactment effects also in free recall (for a review, see [2]; for a review of exceptions, see [41]). However, it is quite unclear what kind of information participants pay attention to during verbal learning. Therefore, using an observation control condition as we did here appears a closer match to the enactment condition. A potentially underestimated functional similarity between enactment encoding and observation could explain why in the majority of studies, similar to the present one, null findings emerged when comparing free recall after enactment encoding and observation in between-subjects designs (e.g. [42]–[46]). Different material factors have been discussed as explanations why in some studies comparable recall levels were found, whereas other studies found an enactment effect (e.g., [26], for an overview). For example, as discussed above, it has been assumed that an enactment effect is more probable for longer lists of unrelated actions where relational processing becomes increasingly difficult [18]. Some studies reported [18] enactment effects in free recall with longer lists of 24 or more actions [7], [18], [26], [47], whereas other studies – similar to the present ones – did not [5], [17], [46]. The reason for this discrepancy in findings remains an open question. In Experiment 1B (but not in Experiment 2), recognition was worse in the action-sequences than the single-actions conditions. This is a strong indicator that the improved recall in the sequences compared to the single-actions conditions was not based on item-specific, but on relational processing. But why would the presence of relations impair item-specific processing? Whereas we have no definite answer, we suspect that the presence of the sequence structure shifted participants' processing from a more detailed to a more global focus. Consequently, when faced with a studied phrase, they apparently found it more difficult to tell whether this was really the presented phrase or a distractor phrase comprising the same object, but a different verb. Whereas we found no interaction with encoding condition, the numbers presented in Table 1 suggest that this was more the case in the observation (recognition difference: −.28) than enactment condition (−.15), in line with the idea that observers of action sequences miss details of individual actions because they take a bird's rather than an ant's perspective (see [5]). In Experiment 2, overall recognition was comparable in the single-actions and the action-sequences conditions. Note, however, that recognition, when assessed after recall, can be influenced by recall performance. Thus, the recognition test in Experiment 1B is a “purer” measure of item-specific processing during study. With regard to the comparison of encoding conditions that is crucial to our purposes, recognition findings in Experiment 2 provide converging evidence with those in Experiment 1B that enactment increases item-specific processing as compared to observation.

**Table 1:** Free Recall Performance (and SD) separately for Encoding Task, List Structure and Recall Test in Experiment 2.

	Single actions			Action sequences		
	Recall test 1	Recall test 2	Recall test 3	Recall test 1	Recall test 2	Recall test 3
Enactment	.21 (.09)	.23 (.09)	.25 (.09)	.24 (.08)	.25 (.08)	.26 (.09)
Observation	.22 (.08)	.23 (.08)	.25 (.07)	.27 (.05)	.26 (.04)	.30 (.04)

## Potential limitations

When planning the above experiments, we chose experimental conditions that we thought most conducive to finding an enactment effect. Bearing in mind that increasing list length could favor enactment encoding, we used long (and very long) lists of simple verb-object phrases. In line with Golly-Häring and Engelkamp [7], who demonstrated an enactment effect for categorical lists based on object similarity, we used lists comprising several action sequences that provided salient relations among action phrases. Analogous to Engelkamp and Zimmer [23], we presented no objects during encoding because symbolic enactment should be sufficient for processing the goal-directed action-related meaning of succeeding action phrases within a sequence. Still, we found no evidence for an enactment effect in free recall, neither in the single-actions condition nor in the action-sequences condition.

However, by choosing these conditions, we neglected several features of real-life activities. For instance, people usually learn one activity at a time instead of several sequences in a row. Similarly, they learn activities in order to carry them out rather than to describe them verbally. Though the present experiments were artificial concerning these aspects, we do not think it severely limits their validity. For instance, Steffens [5] also reported across two experiments comparable memory after enactment and observation in verbal recall as well as reenactment when only one action sequence was learned and when objects were used. Taken together, these findings indicate that also for action-sequence recall, it plays a minor role whether objects are actually presented, or not. Still, we need to concede when interpreting the present findings that the action sequences we used were not identical to those one learns in everyday life (see [48]).

The implementation of the present observation condition may have differed from previous ones. For example, in some studies, participants observed a model on a computer monitor [17], which may introduce a confound as compared to other encoding conditions in which another person is present. Moreover, we instructed participants to observe their vis-à-vis carefully. The actor was another research participants (in almost all cases), whereas in previous studies, it was often the experimenter who performed the actions (e.g.,[49]). Possibly, memory after observation was underestimated in those studies, and our instruction to “carefully observe” increased participants' motivation to pay close attention. Some participants may have interpreted the task of watching their partner's enactment as an instruction to monitor (and evaluate) their partner's pantomime. In a different context, namely, the representation of intentions, it has been demonstrated that such a rather minor difference in instruction affects the pattern of findings [50].

## Conclusion

Given current theorizing on the memory-related consequences of enactment versus observation during study, we had expected clear trade-offs across the measures of memory employed in the present research: As compared to observation, enactment, increasing item-specific processing, should yield advantages in recognition in general as well as in recall of lists of single actions. Conversely, as compared to enactment, observation should increase relational processing and thus lead to better recall of action sequences and better organization of recall protocols. We found an enactment effect in recognition, but none in free recall, and we found weaker evidence than expected for the advantages of observation learning regarding action sequences. In a nutshell, many more similarities than differences in memory after seeing and after doing were found.

## References

[1] Engelkamp J, Cohen RL (1991) Current issues in memory of action events. Psychol Res 53: 175–182.

[2] Engelkamp J (1998) Memory for actions. Hove England: Psychology Press/Taylor & Francis (UK).

[3] Knopf M, Mack W, Lenel A, Ferrante S (2005) Memory for action events: Findings in neurological patients. Scand J Psychol 46: 11–19. 15660629 10.1111/j.1467-9450.2005.00430.x

[4] Engelkamp J, Seiler KH, Zimmer HD (2004) Memory for actions: Item and relational information in categorized lists. Psychol Res 69: 1–10. 14691724 10.1007/s00426-003-0160-7

[5] Steffens MC (2007) Memory for goal-directed sequences of actions: Is doing better than seeing? Psychon Bull Rev 14: 1194–1198. 18229496 10.3758/bf03193112

[6] Engelkamp J, Seiler KH (2003) Gains and losses in action memory. The Quarterly Journal of Experimental Psychology: A 56: 829–848. 10.1080/02724980244000648 12850992

[7] Golly-Häring C, Engelkamp J (2003) Categorical-relational and order-relational information in memory for subject-performed and experimenter-performed actions. J Exp Psychol Learn Mem Cogn 29: 965–975. 14516228 10.1037/0278-7393.29.5.965

[8] Prinz W (1998) Die Reaktion als Willenshandlung. Psychol Rundsch 49: 10–20.

[9] Foley MA, Ratner HH (2001) The role of action-based structures in activity memory. In: Zimmer HD, Cohen RL, Guynn MJ, editors. Memory for action: A distinct form of episodic memory? Oxford: University Press.

[10] Lichtenstein EH, Brewer WF (1980) Memory for goal-directed events. Cogn Psychol 12: 412–445.

[11] Steffens MC, Jelenec P, Mecklenbräuker S (2009) Decomposing the memory processes contributing to enactment effects by multinomial modelling. Eur J Cogn Psychol 21: 61–83.

[12] Kormi-Nouri R (1995) The nature of memory for action events: An episodic integration view. Eur J Cogn Psychol 7: 337–363.

[13] Von Essen JD (2005) Enactment enhances integration between verb and noun, but not relational processing, in episodic memory. Scand J Psychol 46: 315–321. 16014075 10.1111/j.1467-9450.2005.00461.x

[14] Feyereisen P (2009) Enactment effects and integration processes in younger and older adults' memory for actions. Memory 17: 374–385. 19221926 10.1080/09658210902731851

[15] Knopf M (1995) Memory for action events: Structure and development in adulthood. In: Weinert FE, Schneider W, editors. Memory performance and competencies Issues in growth and development. Hillsdale, NJ England: Lawrence Erlbaum Associates, Inc. pp. 127–138.

[16] Steffens MC, Jelenec P, Mecklenbräuker S, Thompson EM (2006) Decomposing retrieval and integration in memory for actions: A multinomial modeling approach. The Q J Exp Psychol 59: 557–576. 10.1080/02724980443000764 16627356

[17] Engelkamp J, Krumnacker H (1980) Imaginale und motorische Prozesse beim Behalten verbalen Materials. Z Exp Angew Psychol 27: 511–533.

[18] Engelkamp J, Dehn DM (2000) Item and order information in subject-performed tasks and experimenter-performed tasks. J Exp Psychol Learn Mem Cogn 26: 671–682. 10855425 10.1037//0278-7393.26.3.671

[19] Hornstein SL, Mulligan NW (2004) Memory for actions: Enactment and source memory. Psychon Bull Rev 11: 367–372. 15260207 10.3758/bf03196584

[20] Manzi A, Nigro G (2008) Long-term memory for performed and observed actions: Retrieval awareness and source monitoring. Memory 16: 595–603. 18569687 10.1080/09658210802070749

[21] Mulligan NW, Hornstein SL (2003) Memory for actions: Self-performed tasks and the reenactment effect. Mem Cognit 31: 412–421. 10.3758/bf03194399 12795483

[22] Koriat A, Ben-Zur H, Druch A (1991) The contextualization of input and output events in memory. Psychol Res 53: 260–270. 10.1007/s004260050034 9870297

[23] Engelkamp J, Zimmer HD (2002) Free recall and organization as a function of varying relational encoding in action memory. Psychol Res 66: 91–98. 12132118 10.1007/s00426-001-0082-1

[24] Roenker DL, Thompson CP, Brown SC (1971) Comparison of measures for the estimation of clustering in free recall. Psychol Bull 76: 45–48.

[25] Engelkamp J, Jahn P, Seiler KH (2003) The item-order hypothesis reconsidered: The role of order information in free recall. Psychol Res 67: 280–290. 14634815 10.1007/s00426-002-0118-1

[26] Engelkamp J, Zimmer HD (1997) Sensory factors in memory for subject-performed tasks. Acta Psychol 96: 43–60.

[27] Ratner HH, Foley MA, McCaskill P (2001) Understanding children's activity memory: The role of outcomes. J Exp Child Psychol 79: 162–191. 11343407 10.1006/jecp.2000.2585

[28] Glover JA, Timme V, Deyloff D, Rogers M (1987) Memory for student-performed tasks. J Educ Psychol 79: 445–452.

[29] Cohen J (1977) Statistical power analysis for the behavioural sciences. Hillsdale, NJ: Erlbaum Associates.

[30] Faul F, Erdfelder E, Lang AG, Buchner A (2007) G*Power 3: A flexible statistical power analysis program for the social, behavioral, and biomedical sciences. Behavior Research Methods 39: 175–191. 17695343 10.3758/bf03193146

[31] Cohen RL, Peterson M, Mantini Atkinson T (1987) Interevent differences in event memory: Why are some events more recallable than others? Mem Cognit 15: 109–118. 10.3758/bf03197022 3683175

[32] Cohen JD, MacWhinney B, Flatt M, Provost J (1993) PsyScope: A new graphic interactive environment for designing psychology experiments. Behavioral Research Methods, Instruments, and Computers 25: 257–271.

[33] Ratner HH, Padgett RJ, Bushey N (1988) Old and young adults' recall of events. Dev Psychol 24: 664–671.

[34] Snodgrass JG, Corwin J (1988) Pragmatics of measuring recognition memory: Applications to dementia and amnesia. J Exp Psychol Gen 117: 34–50. 2966230 10.1037//0096-3445.117.1.34

[35] Tulving E (1962) Subjective organization in free recall of ‘unrelated’ words. Psychological Review 69: 344–354. 13923056 10.1037/h0043150

[36] Klein SB, Loftus J, Kihlstrom JF, Aseron R (1989) Effects of item-specific and relational information on hypermnesic recall. J Exp Psychol Learn Mem Cogn 15: 1192–1197. 2530312 10.1037//0278-7393.15.6.1192

[37] Burns DJ (1993) Item gains and losses during hypermnesic recall: Implications for the item-specific–relational information distinction. J Exp Psychol Learn Mem Cogn 19: 163–173.

[38] McDaniel MA, Moore BA, Whiteman HL (1998) Dynamic changes in hypermnesia across early and late tests: A relational/item-specific account. J Exp Psychol Learn Mem Cogn 24: 173–185. 9438958 10.1037//0278-7393.24.1.173

[39] Payne DG (1987) Hypermnesia and reminiscene in recall: A historical and empirical review. Psychological Bulletin 101: 5–27.

[40] Engelkamp J, Seiler KH, Zimmer HD (2005) Differential relational encoding of categorical information in memory for action events. Mem Cognit 33: 371–379. 10.3758/bf03193055 16156173

[41] Steffens MC, Buchner A, Wender KF, Decker C (2007) Limits on the role of retrieval cues in memory for actions: Enactment effects in the absence of object cues in the environment. Mem Cognit 35: 1841–1853. 10.3758/bf03192919 18265602

[42] Cohen RL (1983) The effect of encoding variables on the free recall of words and action events. Mem Cognit 11: 575–582. 10.3758/bf03198282 6669026

[43] Nadar MS, McDowd J (2008) ‘Show me, don't tell me’; is this a good approach for rehabilitation? Clin Rehabil 22: 847–855. 18728138 10.1177/0269215508091874

[44] Cohen RL, Bean G (1983) Memory in educable mentally retarded adults: Deficit in subject or experimenter? Intelligence 7: 287–298.

[45] Cohen RL (1989) The effects of interference tasks on recency in the free recall of action events. Psychol Res 51: 176–180. 2616696 10.1007/BF00309145

[46] Ratner HH, Hill L (1991) The development of children's action memory: When do actions speak louder than words? Psychol Res 53: 195–202.

[47] Engelkamp J, Zimmer HD (1983) Zum Einfluss von Wahrnehmen und Tun auf das Behalten von Verb–Objekt-Phrasen. Sprache & Kognition 2: 117–127.

[48] Gold DA, Park NW (2009) The effects of dividing attention on the encoding and performance of novel naturalistic actions. Psychol Res 73: 336–349. 18443821 10.1007/s00426-008-0148-4

[49] Cohen RL (1981) On the generality of some memory laws. Scand J Psychol 22: 267–281.

[50] Schult JC, Steffens MC (2011) On the representation of intentions: Do personally relevant consequences determine activation? Mem Cognit 39: 1487–1495. 10.3758/s13421-011-0110-3 21590462

